# Active components of Solanum nigrum and their antitumor effects: a literature review

**DOI:** 10.3389/fonc.2023.1329957

**Published:** 2023-12-19

**Authors:** Han Zhang, Jun-lin Lv, Qiu-sheng Zheng, Jie Li

**Affiliations:** ^1^ School of Integrated Traditional Chinese and Western Medicine, Binzhou Medical University, Yantai, China; ^2^ College of Pharmacy, Shihezi University, Shihezi, China

**Keywords:** *Solanum nigrum*, antitumor, solanine, solamargine, solasonine, solasodine

## Abstract

Cancer poses a serious threat to human health and overall well-being. Conventional cancer treatments predominantly encompass surgical procedures and radiotherapy. Nevertheless, the substantial side effects and the emergence of drug resistance in patients significantly diminish their quality of life and overall prognosis. There is an acute need for innovative, efficient therapeutic agents to address these challenges. Plant-based herbal medicines and their derived compounds offer promising potential for cancer research and treatment due to their numerous advantages. *Solanum nigrum* (*S. nigrum)*, a traditional Chinese medicine, finds extensive use in clinical settings. The steroidal compounds within *S. nigrum*, particularly steroidal alkaloids, exhibit robust antitumor properties either independently or when combined with other drugs. Many researchers have delved into unraveling the antitumor mechanisms of the active components present in *S. nigrum*, yielding notable progress. This literature review provides a comprehensive analysis of the research advancements concerning the active constituents of *S. nigrum*. Furthermore, it outlines the action mechanisms of select monomeric anticancer ingredients. Overall, the insights derived from this review offer a new perspective on the development of clinical anticancer drugs.

## 1 Introduction

Cancer remains a leading cause of mortality worldwide (1). There were an estimated 18.1 million new cancer cases and 9.9 million cancer deaths globally in 2020 (2). According to Cancer Facts & Figures 2023 released by American Cancer Society, over 1.9 million new cancer cases are expected to be diagnosed, and approximately 609,820 deaths from cancer are expected in the US in 2023 (3). Furthermore, global projections indicate that the cancer burden is expected to increase by 50% in 2040 compared with that in 2020, primarily due to an aging population (4). Fortunately, there is promising hope in the battle against cancer, with natural medicines derived from plants and microrganisms offering new avenues of exploration. *Solanum nigrum* (*S. nigrum*), a frequently used herb with antitumor potential in clinical practice, has garnered attention. Within *S. nigrum*, steroidal alkaloids, particularly steroids, represent the crucial chemical constituents responsible for the antitumor properties of *S. nigrum* (5).

Cancer cells are characterized by distinct traits, including sustaining proliferative signals, evading growth inhibitors, resisting programmed cell death, enabling replicative immortality, inducing angiogenesis, promoting metastasis and invasion, remodeling cellular metabolism, and acquiring the ability to evade immune surveillance (6). Patients with primary tumors typically undergo treatments such as surgery, radiotherapy, and chemotherapy (7). Chemotherapeutic agents are cytotoxic drugs commonly used in treating cancer. These drugs target rapidly growing and proliferating cells and promote some of the key factors that impair mitosis and enhance apoptosis during cell division (8). However, chemotherapeutic agents lead to poor prognoses and adverse side effects (9). Common side effects include bone marrow suppression, alopecia (hair loss), fatigue, neuropathy, dermatological issues, and gastrointestinal disorders (10). Traditional Chinese medicine is gradually gaining recognition for its relatively low side effects and antitumor properties.

*S. nigrum*, the largest genus in the Solanaceae family, is an annual herb widely distributed in China (11). It can be used for medicinal purposes, either in its fresh or in its dried form. *S. nigrum* is known for its diverse therapeutic effects, including heat-clearing and toxin-removing properties, reduction of swelling and lumps, anti-inflammatory and diuretic effects, and helping produce saliva and slake thirst (12). In the Ayurvedic tradition of India, *S. nigrum* has been used to treat intestinal diseases, ulcers, diarrhea, and skin conditions (13). Modern pharmacological studies have provided evidence of *S. nigrum*’s antitumor, anti-inflammatory, antioxidant, and antihypertensive properties (14). Furthermore, various active molecules extracted from *S. nigrum* exhibit anticancer effects.

We present a review of the research data from the past decade obtained from PubMed and Web of Science. We conducted a thorough analysis to identify the individual active constituents within *S. nigrum*. Additionally, we explored the antitumor properties and underlying mechanisms of *S. nigrum* in combination with other drugs against different types of tumors.

## 2 Chemical composition of *S. nigrum*

As of 2022, *S. nigrum* has been found to contain a total of 188 chemicals, encompassing steroidal alkaloids, steroidal saponins, glycoproteins, organic acids, lignins, polysaccharides, and polyphenols (15). Among these, steroidal alkaloids and steroidal saponins are the primary active components responsible for its antitumor properties. The steroidal alkaloids in *S. nigrum* primarily consist of three glycosides: solanines, solasonine, and solamargine (SM). These glycosides are primarily present in immature fruits and have been extensively studied in the field of natural products (16).

Predominantly, the alkaloids found in the entire *S. nigrum* herb are steroidal alkaloids, all sharing the fundamental steroidal skeleton structure of cyclopentanoperhydrophenanthrene. This structural motif is currently the subject of widespread research as a potential antitumor active ingredient (17). The fruits of *S. nigrum* are particularly rich in steroidal alkaloids, with the highest content found in the unripe green fruits. As the fruits ripen, their alkaloid composition undergoes changes, leading to a reduction in alkaloid content. As early as 1982, Japanese researchers employed ethanol to isolate two steroidal alkaloids, solasonine and SM, from unripe fruits. These glycosides are linked by glycosidic bonds and share the same glycoside, solasodine (18). Since then, numerous steroidal alkaloids have been identified in *S. nigrum* fruits, including compounds such as 7α-OH-kekasianine, 7α-OH SM, 7α-OH solasonine, 12β,27-dihydroxy solanine-3-O-β-D-glucopyranoside, and 27-solasonine-3-O-β-D-glucopyranosyl-(1→4)-α-L-rhamnopyranoside-(1→2)-[α-L-rhamnopyranoside-(1→4)]-β-D-glucopyranoside (19, 20).

## 3 Pharmacological effects of *S. nigrum*

Modern pharmacological studies have revealed that *S. nigrum* extracts obtained using different solvents yield distinct pharmacological effects. For example, the aqueous extract of *S. nigrum* demonstrates the ability to mitigate angiotensin-II-induced cardiac hypertrophy and improve cardiac health (21). Furthermore, the aqueous extract can inhibit the growth of breast, ovarian, and liver cancer cells by influencing the expression of numerous tumor-related genes (22–30). Additionally, it augments the tumor-inhibitory efficacy of cisplatin, adriamycin, and docetaxel on human ovarian cancer cells (26, 27). Nirmal et al. have demonstrated the antihistaminic and antiallergic effects of the petroleum ether extract of *S. nigrum* fruit, highlighting its potential as a therapeutical agent for asthma (14).

The ethanolic extract of *S. nigrum* has exhibited *in vitro* inhibitory effects on melanoma cells. In *in vivo* experiments, a 1% aqueous extract of *S. nigrum* significantly reduced tumor mass in tumor-bearing mice, achieving a tumor inhibition rate exceeding 50%. Additionally, it effectively inhibited lung metastasis in melanoma (31).

Research on the antitumor effects of individual compounds within *S. nigrum* has predominantly focused on solanine, solasonine, SM, and solasodine. Furthermore, α-solanine has displayed antitumor effects on non–small cell lung cancer (NSCLC) (32). Pharmacological studies have demonstrated that SM exhibits inhibitory effects on gastric, hepatocellular, and lung cancers (33–35). Notably, uttroside B, a saponin isolated from *S. nigrum*, has demonstrated superior anti–liver cancer efficacy over sorafenib, a first-line anticancer drug and antitumor angiogenesis targeted drug (36). Furthermore, uttroside B exhibits fewer toxic effects on normal cells (37). Solasonine has been shown to inhibit pancreatic cancer cells (38). *S. nigrum* has a long history of use in cancer treatment, with its extracts displaying significant antitumor pharmacological activities. Consequently, further research into the isolation and purification of the antitumor active components of *S. nigrum* holds great significance.

**Figure 1:**
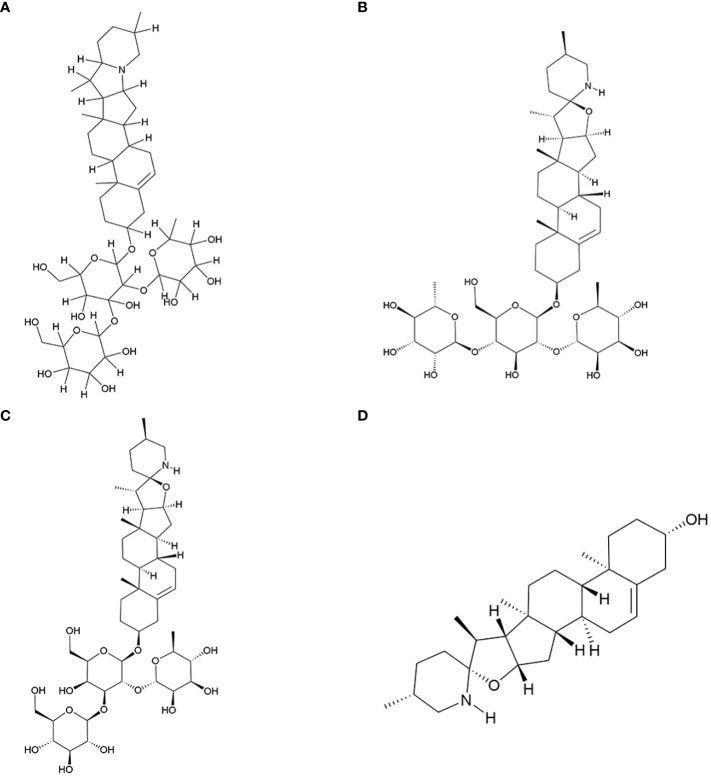
Key antitumor monomer of *S. nigrum*. **(A)** Solanine; **(B)** Solamargine; **(C)** Solasonine; **(D)** Solasodine.

In this study, we have reviewed research conducted over the past two decades on the active components of *S. nigrum* with antitumor properties. Additionally, we have explored the mechanisms underlying the antitumor actions of its four key antitumor monomer active components. The molecular structure of these four components was shown in **Figure 1**.

## 4 Antitumor active monomers from *S. nigrum*

### 4.1 Solanine

Solanine, also referred to as α-solanine, possesses a range of beneficial properties, including antidiabetic, antiallergic, anti-inflammatory, antiviral, antibacterial, antiprotozoan, and antifungal activities. Its anticancer potential is evident through its ability to induce apoptosis and hinder cell growth, migration, and invasion both *in vitro* and *in vivo* (39). Notably, its therapeutic efficacy against liver cancer has been thoroughly investigated.

#### 4.1.1 Anti–liver cancer activity of solanine

Matrix metalloproteinases (MMPs) play a pivotal role in tumor growth and metastasis. Solanine effectively inhibits MMPs, thereby impeding tumor development and stemness. This action involves the downregulation of E-cadherin and upregulation of N-cadherin, resulting in reduced tumor invasiveness and proliferation of HepG2 cells (40). miR-21, a well-established cancer marker, exhibits high expression levels in invasive cancer cells. Remarkably, solanine treatment significantly reduces miR-21 expression, subsequently inhibiting the migration and invasion of liver cancer cells (40). Additionally, solanine intervenes in the immune escape mediated by liver cancer Treg cells through the transforming growth factor (TGF)-β/Smad signaling pathway, ultimately enhancing the immune response (41). In synergy with cisplatin, solanine induces apoptosis, intensifies cell cycle arrest in liver cancer cells, enhances caspase-3 and caspase-7 activity, and attenuates the expression of Bcl-2 and survivin, thereby promoting apoptosis and inhibiting cancer cell growth (42). Furthermore, solanine fosters the expression of ASK1 and TBP-2 and augments their kinase activities by inducing reactive oxygen species (ROS) production in HepG2 cells, ultimately driving apoptosis in these cells (43).

#### 4.1.2 Anti–esophageal cancer activity of solanine

In a study by Wu et al., solanine effectively suppresses the proliferation, migration, and invasion of esophageal cancer cells (EC9706 and Eca109) *in vitro* in a dose- and time-dependent manner. Moreover, solanine triggers apoptosis by activating caspase-3 and caspase-7 and suppresses MMP-2 and MMP-9 expression in a dose-dependent manner. The administration of solanine significantly elevates E-cadherin expression levels, suggesting its ability to inhibit metastasis by influencing cellular protein hydrolysis activation and adhesion capabilities. Solanine treatment also reduces the expression of the apoptosis-related protein Bcl-2, enhances Bax expression, and promotes apoptosis, thus emerging as a potential agent for esophageal cancer prevention and treatment (39). Additionally, solanine downregulates survivin expression by upregulating miR-138 expression, consequently enhancing the radiosensitivity of EC cells and positioning solanine as a promising radiosensitizer (44).

#### 4.1.3 Anti–breast cancer activity of solanine

Solanine treatment is further characterized by increased Bax expression, decreased Bcl-2 levels, and diminished platelet–endothelial cell adhesion molecule (CD31) expression in cancer cells. These actions contribute significantly to anti-angiogenesis, leading to the reduction or disappearance of mammary tumors and the inhibition of tumor progression in mice (45). The anticancer effects of solanine may involve the inhibition of the NF-κB pathway (46). Subsequently, a specialized nanoparticle (DNS) with high solubility capacity was developed. DNS treatment upregulated Bcl-2 expression while downregulating Bax, MMP-2, MMP-9, mTOR, and Akt levels in cancer cells. These findings underscore solanine’s antiangiogenic effect on cancer cells through the activation of the PI3K-Akt pathway (47). Hence, solanine emerges as a promising candidate drug for breast cancer treatment.

#### 4.1.4 Other antitumor activities of solanine

S100P overexpression is known to play a role in promoting tumorigenesis and metastasis in various cancer models (48). S100P is overexpressed in colorectal cancer cells. However, solanine counters this effect by inhibiting S100P expression. Solanine-induced inhibition results in cell cycle arrest at the G0/G1 phase, increased production of ROS, and apoptosis induction in tumor cells. Additionally, solanine has demonstrated its ability to suppress cancer cell proliferation, migration, and stemness by reducing the activity and expression of MMP-2 and MMP-9 (49, 50). Moreover, solanine has been found to elevate ROS levels in lung cancer A549, prostate cancer DU145 cells, and squamous carcinoma KB cells. It activates cellular autophagy by downregulating Akt/mTOR expression (51).

**Figure 2:**
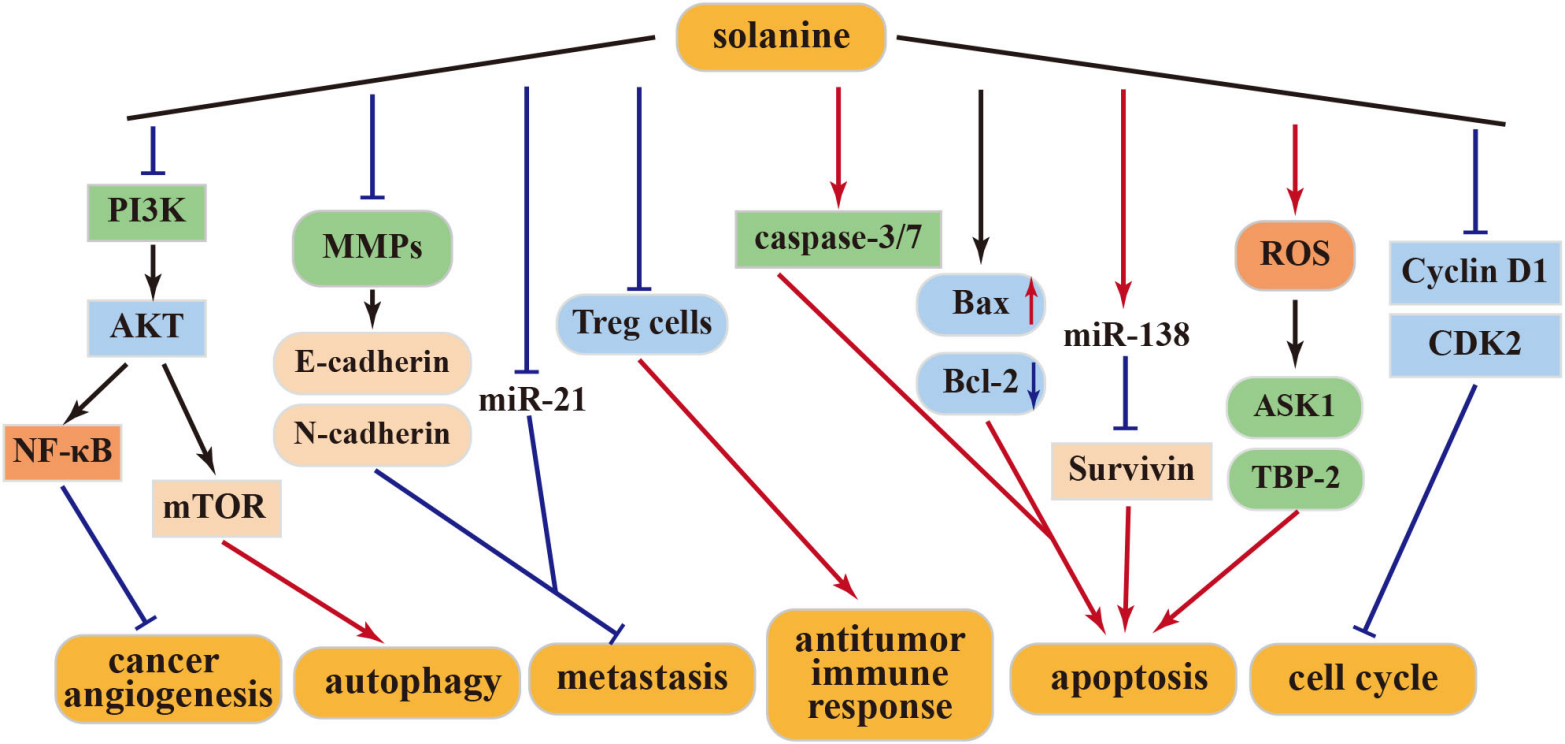
Antitumor mechanism of Solanine.

The above-mentioned studies collectively highlight solanine’s inhibitory effect on various human cancer cells, positioning it as a potential effective antitumor drug in the future. This anticancer activity is attributed to solanine’s ability to modulate different cellular targets, which vary among different cancer cells and at different concentrations. The above antitumor mechanism of solanine was shown in **Figure 2**.

### 4.2 Solamargine

SM is a small-molecule steroidal alkaloid isolated from nightshade plants. Its fundamental chemical structure comprises one glycosidic element linked to three sugar groups through a glycosidic bond, with a molecular formula of C_45_H_73_NO_15_ (52). SM is widely acknowledged as a potent active ingredient in antitumor therapy. SM exhibits robust anticancer activity against various types of cancer, including gastric, lung, liver, and prostate cancers; melanoma; cholangiocarcinoma; and hypopharyngeal squamous cell carcinoma.

#### 4.2.1 Anti–gastric cancer activity of solamargine

SM exerts significant inhibitory effects on gastric cancer cells (33, 53). It induces the expression of caspase-7 and disrupts the G2/M cell cycle to promote cancer cell apoptosis. Furthermore, it upregulates the expression of lncNEAT1_2 and lncPINT by inhibiting the Erk1/2 MAPK signaling pathway, leading to suppressed viability of gastric cancer cells. *In vivo* studies have demonstrated a marked increase in early apoptotic cells and a significant reduction in tumor volume following SM treatment, establishing SM’s potential as a therapeutic agent for gastric cancer (33).

#### 4.2.2 Anti–lung cancer activity of solamargine

In lung cancer, SM acts by inhibiting the expression of SP1 and p65 proteins through the suppression of the PI3K-Akt signaling pathway. This leads to a dose-dependent suppression of the growth of H299 and A549 human lung cancer cells (35). Additionally, SM inhibits DNMT1 protein expression by increasing Erk1/2 phosphorylation, subsequently suppressing c-Jun protein expression and tumor proliferation (54).

HOX transcript antisense RNA (HOTAIR) is often overexpressed in lung cancer and correlates with metastasis and poor prognosis. It promotes the proliferation, survival, invasion, metastasis, and drug resistance of lung cancer cells (55). SM inhibits the proliferation and induces apoptosis of NSCLC cells through inhibition of long-stranded non-coding RNA (HOTAIR). Additionally, SM promotes the expression of miR-214-3p while inhibiting the expression of its downstream target PDPK1. The interplay between HOTAIR and miR-214-3p inhibits NSCLC cell growth (56). Furthermore, the combination of SM and cisplatin exhibits synergistic effects in inhibiting cisplatin-resistant lung cancer cell lines (57), enhancing the antitumor effects of gefitinib and erlotinib (58).

#### 4.2.3 Anti–liver cancer activity of solamargine

Higher expression of Ki67 and PCNA in cells typically indicates increased cell proliferation (59). Treatment of the HepG2 cell line with SM led to reduced expression of Ki67, PCNA, and Bcl-2 proteins while elevating the expression levels of Bax, caspase-3, and caspase-9. As a result, SM is believed to inhibit liver cancer cell proliferation and induce apoptosis by activating the Bcl-2/Bax and caspase signaling pathways (60). Moreover, at high concentrations, SM may effectively treat liver cancer by modulating the LIF/miR-192-5p/CYR61/Akt axis, inducing autophagy and apoptosis. At low concentrations, SM can repolarize M2 macrophages into an M1-like phenotype through LIF/p-Stat3 signaling, inhibiting epithelial–mesenchymal transition (EMT) in hepatoma cells, thereby reducing the invasion and migration abilities of hepatoblastoma cells (34, 61).

MUC1 plays a pivotal role in the transcriptional regulation of genes associated with tumor invasion, metastasis, angiogenesis, proliferation, apoptosis, drug resistance, inflammation, and immune regulation (62–69). According to Tang et al., SM can hinder the growth of hepatocellular carcinoma (HCC) through the HOTTIP-TUG1/miR-4726-5p/MUC1 signaling pathway. Additionally, the combination of SM and sorafenib synergistically inhibits MUC1 protein expression, enhancing the anticancer effect of sorafenib (70).

#### 4.2.4 Other antitumor activities of solamargine

In human castration-resistant prostate cancer (CRPC), SM promotes the phosphorylation of AMPKα and decreases the expression of MUC1 and NF-κB p65 proteins. This leads to the suppression of CRPC cell growth through AMPKα-mediated inhibition of p65 (71). When combined with docetaxel, SM enhances the antitumor effect of docetaxel (72). According to Zhang et al., the treatment of human cholangiocarcinoma cells QBC939 with SM increases the expression of apoptosis-related proteins, inhibiting cell viability. This suggests that SM may induce apoptosis in human cholangiocarcinoma QBC939 cells through the mitochondrial pathway (73).

**Figure 3:**
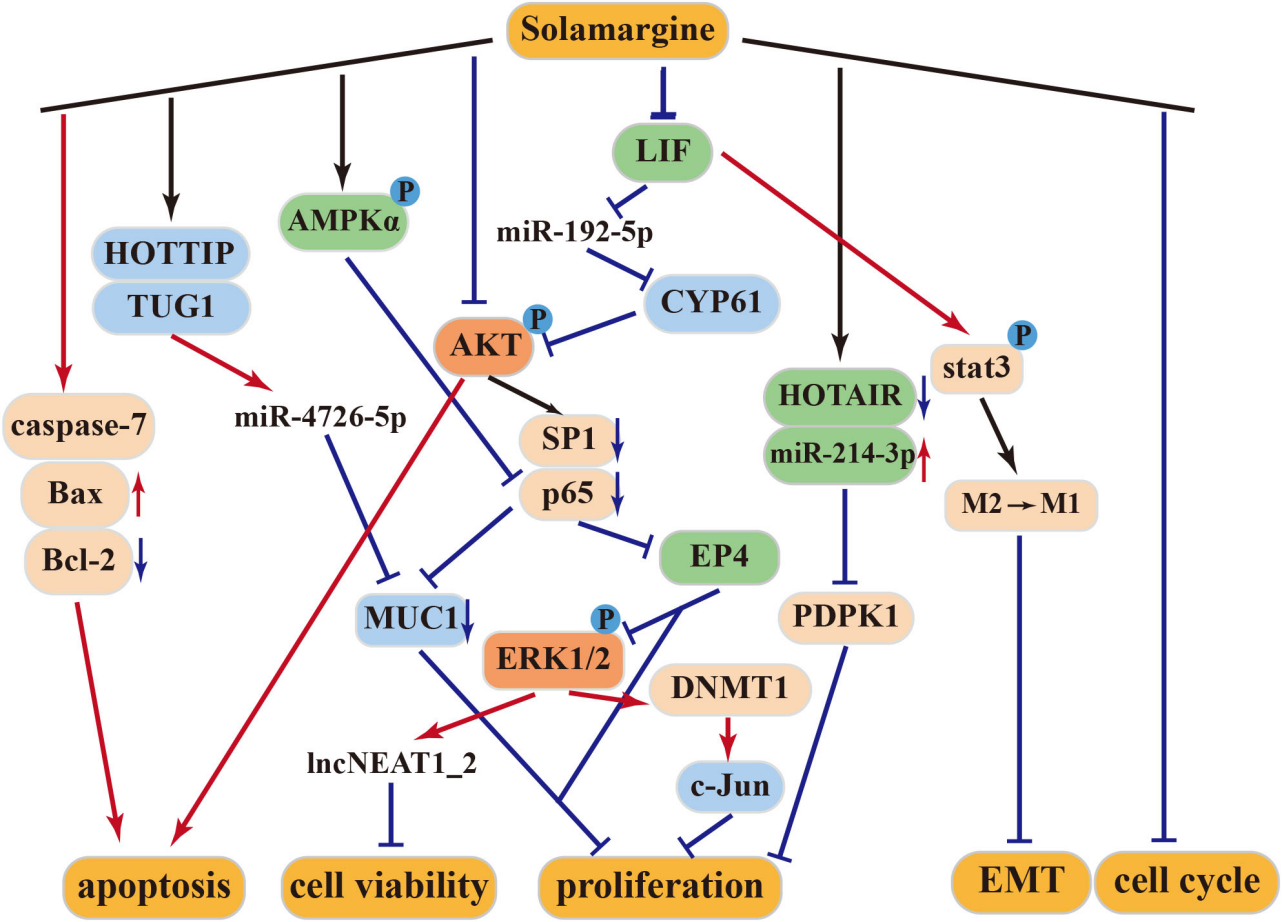
Antitumor mechanism of Solamargine.

SM exerts its antitumor effects through multiple mechanisms, including the tumor suppressor pathway, caspase activation pathway, mitochondrial pathway, apoptosis receptor pathway, protein kinase pathway, and signaling pathways that promote invasion/migration and multidrug resistance (**Figure 3**).

### 4.3 Solasonine

Solasonine, with the molecular formula C_45_H_73_NO_16_, is another typical steroidal alkaloid found in *S. nigrum*. It is commonly used in the treatment of skin diseases and various cancers, and extensive pharmacological studies have been conducted on it.

#### 4.3.1 Anti–liver cancer activity of solasonine

Mortalin, a protein overexpressed in various cancers, sequesters p53 into the cytoplasm, preventing its translocation to the nucleus and inhibiting its cellular functions. Inhibiting the mortalin-p53 interaction is a novel strategy against tumors (74). Solasonine inhibits this interaction, inducing apoptosis in HCC cell lines expressing p53 (HepG2) or in those not expressing p53 (Hep3b). Thus, the apoptotic activity of solasonine can be mediated through both p53-dependent and p53-independent pathways (75). SP1, a direct target of miR-375-3p, can be co-regulated by miR-375-3p and CCAT1. Solasonine can inhibit CCAT1 and SP1 by activating miR-375-3p. Subsequently, IRF5 protein expression is suppressed, inhibiting the proliferation of HepG2 liver cancer cells. Therefore, IRF5 may be a potential target for the treatment of liver cancer (76–79).

#### 4.3.2 Anti-osteosarcoma activity of solasonine

Tumor cells ferment large amounts of glucose into lactic acid even in the presence of oxygen, a phenomenon known as the Warburg effect or aerobic glycolysis, to supply energy (80). Glycolysis is believed to be driven by oncogenic signaling pathways. The Wnt/β-catenin pathway is essential for the development of various embryos. Aberrant activation of this pathway is associated with the development and progression of many human malignancies. Wnt/β-catenin/Snail has been shown to activate EMT, which is closely associated with tumor cell invasion and metastasis (81). According to Wang et al., solasonine upregulates E-cadherin expression and downregulates N-cadherin expression, inhibiting EMT in cells and effectively restraining the migration and invasion of osteosarcoma cells. This inhibition of aerobic glycolysis in osteosarcoma cells, along with reduced lactate secretion and glucose consumption and increased ROS production in cell supernatants, is mediated by solasonine through the Wnt/β-Catenin/Snail pathway in an ALDOA-dependent manner. These effects inhibit cancer cell proliferation and migration in both *in vivo* and *in vitro* settings (82).

#### 4.3.3 Other antitumor activities of solasonine

**Figure 4:**
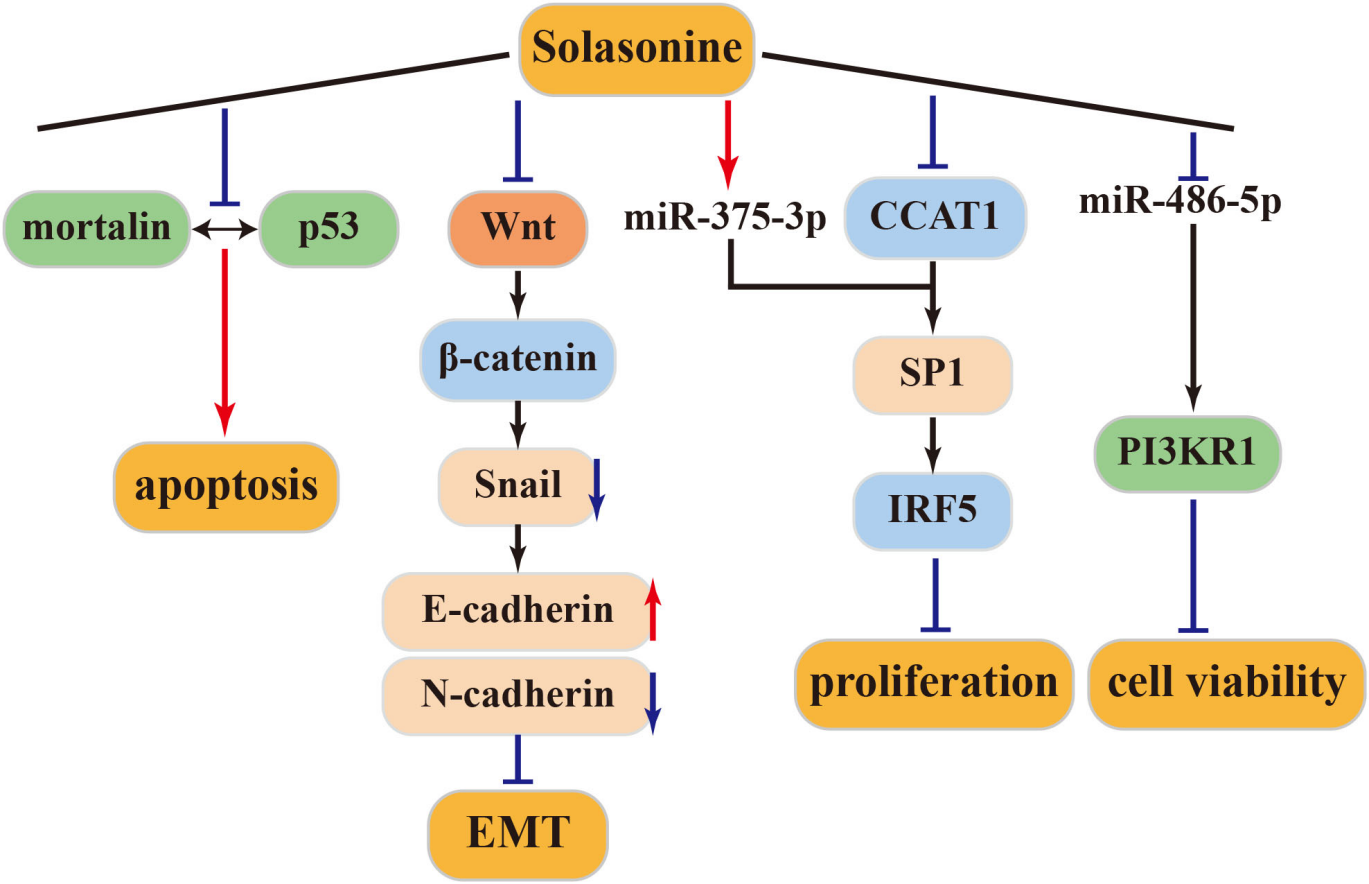
Antitumor mechanism of Solasonine.

Solasonine, either alone or in combination, can trigger apoptosis in the SGC-7901 human gastric cancer cell line through the mitochondrial apoptosis pathway. Furthermore, when combined with cisplatin, solasonine enhances cisplatin’s activity, resulting in improved therapeutic outcomes (83). Moreover, solasonine is effective at inhibiting cancer cell proliferation and increasing sensitivity to chemotherapeutic drugs by targeting miR-486-5p, thereby increasing its expression. miR-486-5p, which post-transcriptionally regulates PI3KR1, plays a regulatory role in gastric cancer. These findings underscore solasonine ‘s antigastric cancer effects by modulating the miR-486-5p/PI3KR1 axis (84). The above antitumor mechanism of solasonine was shown in **Figure 4**.

### 4.4 Solasodine

Solasodine, with a molecular formula of C_27_H_43_NO_2_, is a steroidal alkaloidal sapogenin known for its pharmacological effects such as antioxidant properties (85) and anticonvulsant activity (86). Many studies have highlighted the potent cytotoxicity of solasonine against various types of tumor cells, which is described in the subsequent sections.

#### 4.4.1 Anti–pancreatic cancer activity of solasodine

In clinical treatment, solasodine (CTX) is commonly employed due to its potent anticancer activity against pancreatic cancer (87). In a SW1990 tumor-bearing mouse model, solasodine exhibited superior efficacy in inhibiting tumor growth over CTX, while exerting lower toxicity to normal cells. Solasodine achieved this by inhibiting the Cox-2/Akt/GSK3β signaling pathway in pancreatic cancer cells, leading to increased expression of cyt-C, caspase-9, caspase-3, and Bax and decreased expression of Bcl-2 in a dose-dependent manner. Additionally, solasodine stimulated the immune response, significantly elevating serum levels of TNF-α, IL-2, and IFN-γ in tumor-bearing mice *in vivo* (88).

#### 4.4.2 Anti–gastric cancer activity of solasodine

Solasodine demonstrated a dose-dependent downregulated expression of caspase-3, caspase-7, caspase-9, and poly-ADP-ribose polymerase (PARP) in GC cells HGC27. It also reduced the levels of GPX4 and SLC7A11, although it did not alter the levels of ROS and malondialdehyde. Moreover, solasodine promoted apoptosis and ferroptosis in cancer cells, effectively inhibiting tumor growth. Through the inhibition of the RhoA/STAT 3/NF-κB pathway and reduction of CLDN 2 through the AMPK pathway, solasodine suppressed metastasis and inhibited EMT in GC cells. Hence, solasodine presents itself as a potential antitumor drug for GC (89).

#### 4.4.3 Anti–colorectal cancer activity of solasodine

Solasodine can inhibit human colorectal cancer cells by targeting the AKT/GSK-3β/β-catenin axis and inducing apoptosis in rectal cancer cells through the activation of the caspase cascade (90). Treatment of rectal cancer HCT 116 cells with varying concentrations of solasodine resulted in reduced protein levels of tumor stemness markers, including CD133, CD44, Nanog, Oct-4, and Sox-2. Additionally, these treatments inhibited TGF-β1-induced invasion and migration of HCT 116 cells. These results suggest that solasodine can reverse the stemness of colorectal cancer cells *in vitro* and *in vivo*, while also suppressing the metastasis and invasion of cancer cells, thereby exerting its anticancer effects (91).

**Figure 5:**
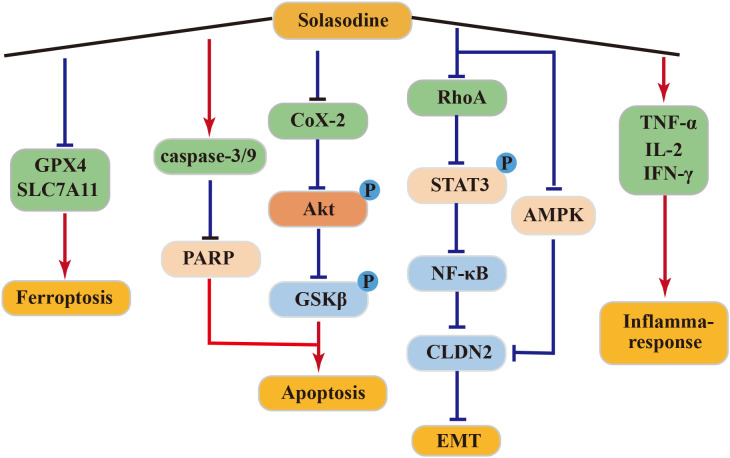
Antitumor mechanism of Solasodine.

In conclusion, solasodine exhibits potent antitumor effects and holds promise as a potentially active drug for cancer treatment. The above antitumor mechanism of solasonine was shown in **Figure 5**.

## 5 Future prospects

Traditional Chinese medicine has emerged as a significant strategy for cancer treatment, offering a novel therapeutic approach characterized by its ability to target multiple components, affect various signaling pathways, and reduce side effects. *Astragalus membranaceus*, *S. nigrum*, Lotus plumule, and *Ligusticum wallichii* are prominent traditional Chinese medicines widely utilized in clinical antitumor treatments. Due to its inherent toxicity, *S. nigrum* is frequently combined with other drugs in clinical settings, resulting in remarkable antitumor effects, substantial medicinal value, and promising prospects for further development. In recent years, fewer in-depth studies have explored the pharmacological mechanisms of the active monomer components within *S. nigrum*. Therefore, future research should prioritize investigating this aspect. Combining individual components with traditional chemotherapeutic drugs such as solanine, SM, and cisplatin, has proven to enhance tumor suppression efficiency and mitigate drug resistance in tumor cells. This innovative approach opens up a fresh avenue for investigating the anticancer properties of *S. nigrum*. Notably, a series of derivatives have recently been identified based on the active monomer components, demonstrating significant anticancer pharmacological activities. The development of new derivatives and targeted dosage forms also holds substantial research potential.

## 6 Conclusion

This study provides a comprehensive review of the four anti-tumor active components found in the Chinese medicinal herb *S. nigrum*, along with their molecular mechanisms of action (**Table 1**). *S. nigrum* is widely utilized in clinical Chinese medicine and traditional folk Chinese herbal formulas. A wealth of evidence supports the notion that the active components in *S. nigrum* can effectively inhibit cancer cells, reverse drug resistance in tumors, and reduce the stemness of tumor stem cells. The antitumor effects and mechanisms of *S. nigrum* primarily encompass the inhibition of cell proliferation, cell cycle arrest, induction of apoptosis, suppression of EMT and tumor metastasis, reversal of drug resistance, and enhancement of the efficacy of radiotherapy and targeted therapy. This review delves into the antitumor mechanisms of the active monomer components in *S. nigrum*, offering valuable theoretical insights for the rational utilization and further development of *S. nigrum* in cancer treatment.

**Table 1:** Antitumor mechanism of four key components of *S. nigrum*.

Compounds	Subjects (cells/animals)	Concentration	Safe dose for animals (mg/kg)	Research mechanisms	Tumor type	References
Solanine	HepG2, H22, Hepatoma patients	0-20 μM	37.5	MMP-2, MMP-9 synthetic signaling pathway, Synthetic signaling pathway, miR-21, TGFβ/Smad signaling pathway, inhibition of Treg cells, Caspase pathway, ROS pathway	Hepatoma	[36-39]
	EC9706, KYSE30	0-6 μM	0-3.5	miR-138, Caspase pathway	Esophageal cancer	[35, 40]
	4T1	0-50 μM	0-100	Apoptosis pathway	Breast cancer	[41-43]
	SW480, SW620, HT-29, RKO, HCT116	0-32 μM	5-10	MMP-2, MMP-9 synthetic signaling pathway, Caspase pathway, Apoptosis, ROS pathway, S100P	Colorectal cancer	[45, 46]
Solamargine	H1650, H1975, PC9, A549, H1299	0-6 μM	4-8	Inhibition of prostaglandin E2, c-Jun signaling pathway, HOTAIR	Lung cancer	[50, 52]
	SMMC7721, HepG2	0-20 μM	/	Bcl-2/Bax and caspase pathway, LIF/miR-192-5p/CYR61/Akt, LIF/p-Stat3, Suppression of MUC1 gene expression	Hepatoma	[30, 56, 57]
	DU145, PC3	0-10 μM	5-10	Suppression of MUC1 gene expression, p56 signaling pathway	Prostate cancer	[67]
	QBC939	0-10 μM	/	Apoptosis pathway	Cholangiocarcinoma	[69]
Solasonine	HepG2, Hep3b, QGY7703, LO2	0-50 μM	10-100	mortalin-p53 signaling pathway, miR-375-3p	Hepatocellular carcinoma	[71-75]
	HOS, U2OS	0-40 μM	0-50	Wnt/β-Catenin/Snail pathway	Osteosarcoma	[89]
	SGC-7901, SNU1, SNU5	0-40 μM	/	Mitochondrial pathway, Endoplasmic reticulum stress pathway, Caspase pathway, miR-486-5p/PI3KR1 axis	Gastric cancer	[79,80]
Solasodine	SW1990, PANC1	0-40 μg/μL	0-15700	f Cox-2/Akt/GSK3β signal pathway, Caspase pathway, Stimulating immunity	Pancreatic Cancer	[83]
	HGC27, SGC7901, NCI–N87, AGS	0-10 μM	20	AMPK/STAT3/NF-κB/CLDN2 signalling pathway	Gastric cancer	[85]
	HCT16, HT-29, SW480	10-80 μM	30-50	MMP-2, MMP-9, MMP-14 synthetic signaling pathway, AKT/GSK-3b/b-catenin signaling pathway	Colorectal cancer	[86, 87]

## Author contributions

HZ: Writing – original draft. J-LL: Writing – original draft. Q-SZ: Conceptualization, Project administration, Supervision, Writing – review & editing. JL: Conceptualization, Project administration, Supervision, Writing – review & editing.
